# Atorvastatin attenuates ferroptosis-dependent myocardial injury and inflammation following coronary microembolization *via* the Hif1a/Ptgs2 pathway

**DOI:** 10.3389/fphar.2022.1057583

**Published:** 2022-12-08

**Authors:** Tao Liu, Jin Shu, Yangchun Liu, Jian Xie, Tao Li, Haoliang Li, Lang Li

**Affiliations:** ^1^ Department of Cardiology, The First Affiliated Hospital of Guangxi Medical University, Nanning, China; ^2^ Cardiothoracic Surgery Intensive Care Unit, The First Affiliated Hospital of Guangxi Medical University, Nanning, China; ^3^ Department of Cardiology, The Second Affiliated Hospital of Anhui Medical University, Hefei, China; ^4^ Guangxi Key Laboratory of Precision Medicine for Prevention and Treatment of Cardiovascular and Cerebrovascular Diseases, Nanning, China

**Keywords:** coronary microembolization, ferroptosis, inflammation, atorvastatin, myocardial injury

## Abstract

**Objectives:** Coronary microembolization (CME) represents a serious periprocedural complication after percutaneous coronary intervention. Ferroptosis has been identified in multiple cardiovascular diseases. In this study, we aimed to investigate the effects of atorvastatin (ATV) on ferroptosis and inflammation following CME and elucidate the underlying mechanism.

**Methods:** We established a rat model of CME by injecting microspheres into the left ventricle. Deferoxamine (DFO), a selective ferroptosis inhibitor, or ATV was pretreated before modeling. Cardiac function and cardiac troponin T (cTnT) levels were detected. Levels of ferroptosis-associated genes, malondialdehyde (MDA), glutathione (GSH), and ferrous iron (Fe^2+^) were measured to validate ferroptosis. Levels of tumor necrosis factor alpha (TNF-α) and interleukin 1 beta (IL-1β) were assayed to determine the inflammation. Chromatin immunoprecipitation was performed to determine the binding of hypoxia-inducible factor 1 subunit alpha (Hif1a) to the promoter of prostaglandin-endoperoxide synthase-2 (Ptgs2).

**Results:** Ferroptosis and inflammation were induced following CME with increased levels of MDA (∼2.5 fold, *p* < 0.01), Fe^2+^ (∼1.5 fold, *p* < 0.01), TNF-α, and IL-1β and decreased GSH levels (∼42%, *p* < 0.01). Meanwhile, the level of Ptgs2 was significantly increased, while those of glutathione peroxidase 4 (Gpx4) and solute carrier family 7 member 11 (Slc7a11) were decreased. The level of cTnT was increased by 7-fold (*p* < 0.01). Left ventricular ejection fraction (LVEF) was significantly reduced (∼85% in the sham group *versus* ∼45% in the CME group, *p* < 0.01). DFO or Ptgs2 silencing inhibited the increase of MDA, Ptgs2, TNF-α, and IL-1β, and induced the levels of GSH and Gpx4, followed by reduction in cTnT levels by approximately 50% (*p* < 0.01). LVEF was improved by approximately 2 fold (*p* < 0.01). Mechanistically, the transcription factor Hif1a bound to the promoter of Ptgs2 and upregulated its expression. In addition, ATV inhibited the activation of the Hif1a/Ptgs2 axis and attenuated cardiac ferroptosis and inflammation, thus ameliorating CME-induced myocardial injury (LVEF, ∼34% elevation; cTnT, ∼1.8 fold decrease, *p* < 0.01).

**Conclusion:** Atorvastatin ameliorates ferroptosis-mediated myocardial injury and inflammation following CME *via* the Hif1a/Ptgs2 pathway.

## Introduction

Coronary microembolization (CME), resulting from atherosclerotic plaque debris and thrombotic materials, is one of the main causes of the “no-reflow” phenomenon (Kleinbongard and Heusch, 2022). The incidence of CME during percutaneous coronary intervention (PCI) has been reported to be between 0% and 70%, depending on its assessment methods (Heusch et al., 2009). CME induces microinfarcts and an inflammatory response along with decreased coronary reserve and cardiac marker elevation, which contributes to progressive myocardial dysfunction (Herrmann et al., 2001; Kleinbongard and Heusch, 2022). Numerous studies have indicated that CME represents a potent predictor of adverse outcomes. The application of protection devices to retrieve plaque debris and thrombotic materials has not been demonstrated to improve the clinical prognosis. The prevention and treatment of CME are mostly dependent on antithrombotic, vasodilator, and anti-inflammatory agents (Kleinbongard and Heusch, 2022). Therefore, it is crucial to develop potent interventions for the prevention and treatment of CME.

Ferroptosis is a novel form of programmed cell death characterized by the iron-mediated accumulation of lipid peroxidation (Stockwell, 2022). Ferroptosis has been identified in multiple cardiovascular diseases, such as myocardial ischemia-reperfusion injury, diabetic cardiomyopathy, and heart failure (Fang et al., 2022). Mounting research has demonstrated the crucial role of ferroptosis in the inflammatory response, and several anti-inflammatory agents function as ferroptosis inhibitors (Sun et al., 2020). Previous research demonstrated that inflammation contributed to CME-induced myocardial injury, in which TNF-α plays a crucial role (Dörge et al., 2002; Thielmann et al., 2002). However, the function and underlying mechanism of ferroptosis and inflammation in CME-induced myocardial injury are largely unclear.

The enzyme prostaglandin-endoperoxide synthase-2 (Ptgs2) is pivotal in modulating the expression levels of prostaglandins, which are the master mediators of the inflammatory response (Tsatsanis et al., 2006). The genetic decrease in Ptgs2 has been reported to be associated with a lower risk of cardiovascular diseases (Ross et al., 2014). In addition, an increasing number of studies have demonstrated that Ptgs2 is a valid marker of ferroptosis (Li et al., 2019; Fang et al., 2020). However, its role in ferroptosis and inflammation following CME is still unknown.

Hypoxia-inducible factor 1 subunit alpha (Hif1a) is a transcriptional activator that functions as a master regulator in maintaining oxygen homeostasis (Hölscher et al., 2012). Mounting evidence has revealed that Hif1a transcriptionally modulates the expression of multiple genes and plays a bidirectional role in regulating cellular functions. However, few studies have focused on the effects of Hif1a on ferroptosis-dependent myocardial injury and inflammation following CME.

Atorvastatin (ATV) remains the cornerstone of pharmacological treatment for atherosclerotic cardiovascular diseases (Visseren et al., 2021). Periprocedural statins administration has been determined to be effective in attenuating myocardial injury following elective coronary intervention (Herrmann et al., 2002; Briguori et al., 2004; Berwanger et al., 2018). Our previous studies demonstrated that ATV pretreatment ameliorated myocardial dysfunction following CME (Li et al., 2011). However, the underlying protective mechanism of ATV on CME-induced myocardial injury has not been elucidated.

In the present study, we hypothesize that ATV could attenuate ferroptosis-dependent myocardial injury and inflammation following CME by inhibiting the Hif1a/Ptgs2 pathway.

## Materials and methods

### Animal model of CME and *in vivo* treatment

Adult male Sprague–Dawley rats (approximately 250 g) were obtained from the Animal Center of Guangxi Medical University (Nanning, China). The protocol was approved by the Animal Care and Use Committee of Guangxi Medical University. The investigation conformed to the Guide for the Care and Use of Laboratory Animals published by the National Academies Press (8th edition, revised 2011).

The CME model was established as previously described (Dörge et al., 2000; Li et al., 2010; Zhou et al., 2021). Briefly, rats were anesthetized intraperitoneally with 3% pentobarbital sodium (40 mg/kg). After mechanical ventilation, the rats were subjected to a thoracotomy to expose the hearts and ascending aorta. Finally, the ascending aorta was occluded for 10 s, and 8,000 polyester microspheres (diameter 42 μm, Biosphere Medical, Rockland, MA, United States ) were injected into the left ventricle at the same time, while the sham-operated rats received the same dosage of normal saline instead. For *in vivo* treatment, rats were treated with recombinant adeno-associated virus 9 (rAAV9)-GFP-shPtsg2 (Hanbio, Shanghai, China) or rAAV9-GFP-shHif1a (Genechem, Shanghai, China) at a dose of 1 × 10^12^ VG in 200 µL saline *via* a single tail vein before CME. Deferoxamine (DFO) was purchased from Selleck (Shanghai, China) and administered intraperitoneally at a dose of 100 mg/kg for 7 days before CME. ATV (Pfizer, New York, United Kingdom) was administered intragastrically at a dose of 10 mg/kg for 7 days before CME. The vehicle control received an administration of an equal volume of saline instead. The rats were sacrificed at 12 h after surgery. The shRNA sequences are listed in Table 1.

**TABLE 1:** shRNA used in the study.

shRNA	Sense (5’-3’)	Antisense (5’-3’)
shNC	UUC​UCC​GAA​CGU​GUC​ACG​U TT	ACG​UGA​CAC​GUU​CGG​AGA​A TT
shPtgs2	AGA​UAG​UGA​UCG​AAG​ACU​ACG TT	UAG​UCU​UCG​AUC​ACU​AUC​UUG TT
shHif1a	GAG​CCT​TAA​CCT​ATC​TGT​CAC	GTG​ACA​GAT​AGG​TTA​AGG​CTC

### Echocardiography

An ESAOTE MyLab system (ESAOTE, Italy) was utilized to measure cardiac function. The left ventricular end-systolic diameter (LVESD) and left ventricular end-diastolic diameter (LVEDD) were measured from three consecutive cardiac cycles. The left ventricular ejection fraction (LVEF) and fractional shortening (FS) were calculated using the following formula: LVEF (%) = [(LVEDV - LVESV)/LVEDV] × 100%, FS(%) = [(LVEDD - LVESD)/LVEDD]×100%. LVESV: left ventricular end-systolic volume; LVEDV: left ventricular end-diastolic volume.

### Cardiac biomarker detection

The level of cardiac troponin T (cTnT) in serum samples was assayed using an ELISA kit (Bioswamp, Wuhan, China) following the manufacturer’s instructions.

### Real-time quantitative polymerase chain reaction

Total RNA was extracted from heart tissues using TRIzol (Invitrogen, United States ). A NanoDrop 2000 was utilized to examine the purity and quantity of RNA. First-strand cDNA was synthesized using HyperScript III SuperMix for qPCR with gDNA Remover (EnzyArtisan, Shanghai, China). RT‒qPCR was performed in 10 µL reactions on an Applied Biosystems 7,500 System (Applied Biosystems, United States ) using 2 × S6 Universal SYBR Green qPCR Mix (EnzyArtisan, Shanghai, China). The relative expression levels normalized to GAPDH were calculated by the 2^−ΔΔCT^ method. All primer sequences used for RT‒qPCR are listed in Table 2.

**TABLE 2:** Primer used in the study.

Gene/Segment	Forward	Reverse	Amplification length (bp)
Gpx4	AGC​AAG​ATC​TGT​GTA​AAT​GGG	TTT​GAT​GGC​ATT​TCC​CAG​C	95
Ptgs2	CCA​ACC​TCT​CCT​ACT​ACA​CC	CCT​TAT​TTC​CTT​TCA​CAC​CCA	80
IL-1β	AAC​TGT​GAA​ATA​GCA​GCT​TTC​G	CTG​TGA​GAT​TTG​AAG​CTG​GAT​G	81
TNF-α	AGG​AGG​GAG​AAC​AGC​AAC​TC	TGT​ATG​AGA​GGG​ACG​GAA​CC	93
Hif1a	TTT​GCA​GAA​TGC​TCA​GAG​G	TGC​AGT​AAC​GTT​CCA​ATT​CC	80
GAPDH	GAC​ATG​CCG​CCT​GGA​GAA​AC	AGC​CCA​GGA​TGC​CCT​TTA​GT	92
P1	TTG​CCA​TAG​CAT​ATC​TTC​TTG​TA	CTC​CCT​CTG​TTA​CAT​AGC​TT	144
P2	CAA​TGC​GGT​GGA​CAC​TTA​GC	TTT​CAC​CGA​ACT​GTC​CTC​CA	111
P3	AAC​TCC​ACC​AAT​GCA​GAT​GTC	CGG​AGG​AGC​AAG​AGA​ATG​TC	203
P4	GCG​GAA​AGA​CAC​AGT​CAC​GA	CTT​CGT​AGG​CAG​GGT​CTT​CG	131

Gpx4, glutathione peroxidase 4; Ptgs2, prostaglandin-endoperoxide synthase-2; IL-1β, interleukin 1 beta; TNF-α, tumor necrosis factor alpha; Hif1a, hypoxia-inducible factor 1 subunit alpha.

### Western blotting

Total protein from heart tissues was extracted using RIPA lysis buffer (Beyotime, Shanghai, China). A BCA protein assay kit (Beyotime, Shanghai, China) was used to measure the protein concentration. Protein samples (30 µg) were fractionated on 10% SDS‒PAGE gels and transferred onto PVDF membranes (GE Healthcare Life Sciences, Boston, United States ). The membranes were blocked with 5% skim milk for 1 h at room temperature and then incubated with the corresponding primary antibodies at 4°C overnight. The primary antibodies were as follows: Hif1a (Wanleibio, WL01607, 1:500), Ptgs2 (Proteintech, 27308-1-AP, 1:1,000), Gpx4 (Abcam, ab125066, 1:5,000), NRF2 (Abcam, ab92946, 1:1,000), Slc7a11 (Abcam, ab175186, 1:1,000), IL-1β (Wanleibio, WL00891, 1:500), TNF-α (Wanleibio, WL01581, 1:1,000) and GAPDH (Abcam, ab181602, 1:10,000). The membranes were incubated with an HRP-conjugated secondary antibody at room temperature for 1 h. Finally, the bands were detected by the FluorChem E System (ProteinSimple, United States ) using an enhanced chemiluminescence kit (Biosharp, Hefei, China). The protein intensity was determined by ImageJ software (NIH, United States ). GAPDH served as a loading control and all data were quantitatively normalized to the GAPDH.

### Immunohistochemical staining

The procedure was previously described (Liu et al., 2015). Briefly, sections were deparaffinized, hydrated, and followed by antigen retrieval. Then the sections were incubated with CD45 antibody followed by staining with HRP polymer conjugated secondary antibody. Images were obtained using BX53F microscope (Olympus, Japan).

### Malondialdehyde and glutathione assays

A lipid peroxidation MDA assay kit (Beyotime, Shanghai, China) was used to determine the relative MDA concentrations in myocardial tissues following the manufacturer’s protocol.

The relative concentrations of GSH in myocardial tissues were determined using a GSH assay kit (Beyotime, Shanghai, China) according to the manufacturer’s instructions. The absorbance was immediately measured at 412 nm on a VARIOSKAN LUX microplate reader (Thermo Fisher Scientific, United States ).

### Iron assay

Heart tissue (10 mg for each sample) was washed with prechilled PBS and homogenized. After centrifugation, the supernatant was collected for detection. Intracellular ferrous iron (Fe^2+^) levels were determined using an Iron Assay Kit (ab86633, Abcam, United Kingdom) according to the manufacturer’s instructions. The absorbance at 593 nm was measured using a VARIOSKAN LUX microplate reader (Thermo Fisher Scientific, United States ).

### Chromatin immunoprecipitation qPCR analysis

The ChIP assay was performed using the EZ-Magna ChIP kit (EMD Millipore, Germany) according to the manufacturer’s instructions. The primer sequences used for ChIP‒qPCR are listed in Table 2.

### Transmission electron microscopy

Fresh myocardial tissues were prefixed in 2.5% glutaraldehyde and fixed in 1% osmium tetroxide. Then, the samples were dehydrated in ethanol with 3% uranyl acetate, embedded in epoxy resin and propylene oxide overnight, and polymerized. Sections were sliced into 70-nm-thick sections and stained with lead citrate. The images were captured by an H-7650 transmission electron microscope (Hitachi, H-7650, Japan).

### Data normalization

Data were normalized to the control. Briefly, we averaged all values in the control to calculate a mean. Next, the mean value was divided by all values in the control, which makes the control one.

### Statistical analysis

All values are presented as the mean ± SEM (standard error of the mean). Student’s t test or Mann-Whitney test was applied to assess the variations between two independent groups. One-way ANOVA was used for comparisons among multiple groups, followed by the Tukey test for further *post hoc* analysis. Statistical analysis was performed using SPSS 19.0 statistical software (IBM Corporation, United States ). A *p*-value <0.05 was considered statistically significant.

## Results

### Ferroptosis and inflammation were involved in CME-induced myocardial injury

**FIGURE 1:**
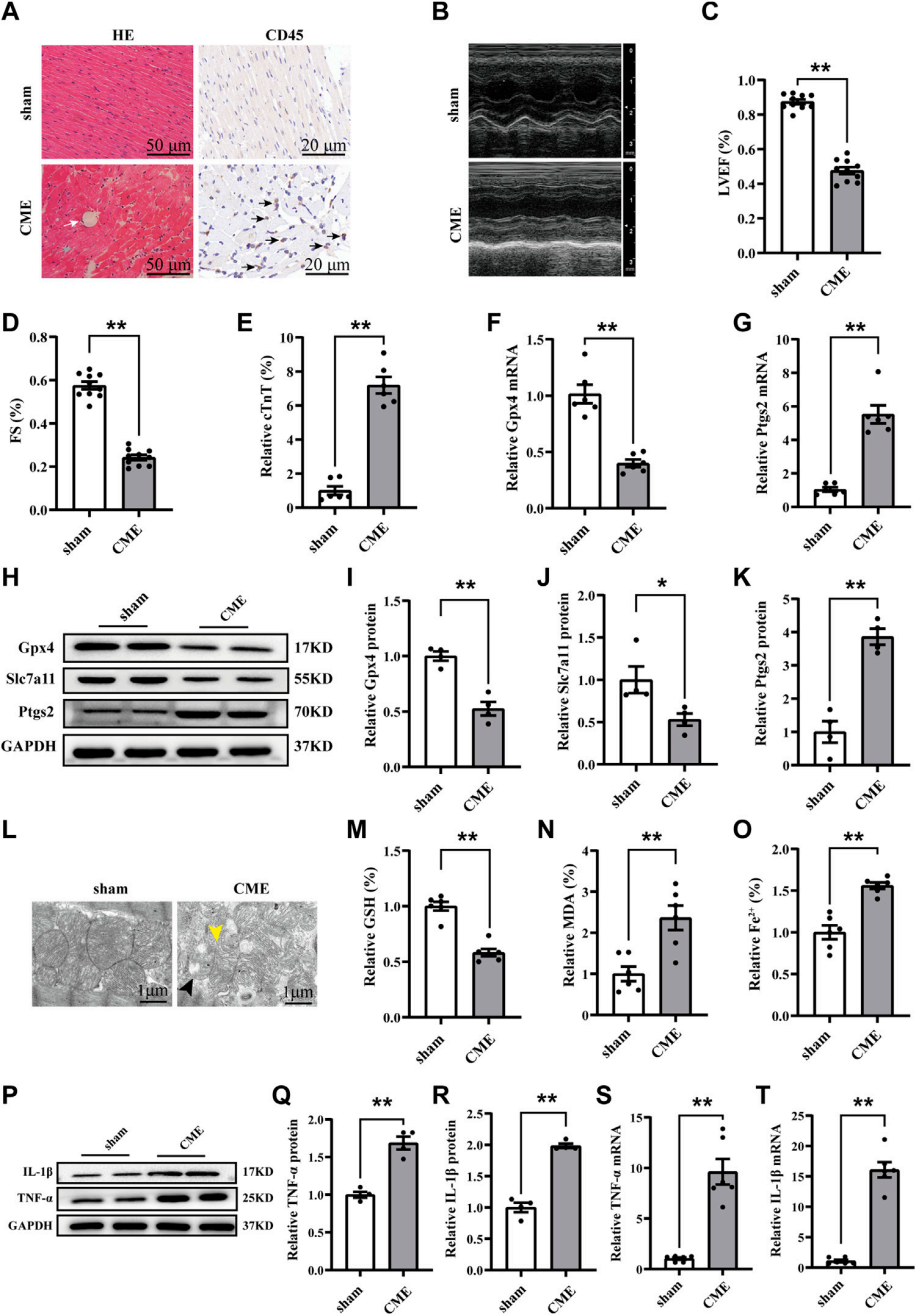
Ferroptosis and inflammation involved in CME-induced myocardial injury. **(A)** Myocardial tissues stained with H&E (scale bar = 50 μm) and immunohistochemistry (scale bar = 20 μm). The white arrow indicates microspheres. The gray arrow indicates microinfarcts. The black arrow indicates the infiltration of inflammatory cells. **(B–D)** Cardiac LVEF and FS detected by echocardiography (*n* = 10). **(E)** Serum levels of cTnT in the sham and CME group (*n* = 6). **(F,G)** The expression levels of Gpx4 and Ptgs2 mRNA (*n* = 6). **(H–K)** Western blotting analysis of Gpx4, Slc7a11, and Ptgs2 (*n* = 4). **(L)** Representative images of transmission electron microscopy. The black arrow indicates the reduced or even disappeared mitochondrial cristae. The yellow arrow indicates the rupture of the outer membrane. Scale bar = 1 μm. **(M–O)** Cardiac levels of GSH, Fe^2+^, and MDA were measured (*n* = 6). **(P–T)** The expression of IL-1β and TNF-α in mRNA (*n* = 6) and protein levels (*n* = 4). GAPDH served as an internal control and was performed to quantitatively normalized the protein data. Data are presented as the normalized mean ± SEM (to sham) or mean ± SEM. Values in shams were averaged and normalized to 1 **(E–T)**. *p < 0.05. **p < 0.01. CME: coronary microembolization; H&E: hematoxylin and eosin; LVEF: left ventricular ejection fraction; FS: fractional shortening; cTnT: cardiac troponin T; Gpx4: glutathione peroxidase 4; Slc7a11: solute carrier family 7 member 11; Ptgs2: prostaglandin-endoperoxide synthase-2; MDA: malondialdehyde; GSH: glutathione; IL-1β: interleukin 1 beta; TNF-α: tumor necrosis factor alpha.

Microinfarcts and the infiltration of inflammatory cells were detected following CME (Figure 1A). Myocardial function was decreased, and the level of the cardiac biomarker cTnT was increased following CME (Figures 1B–E). The expression of glutathione peroxidase 4 (Gpx4) and solute carrier family 7 member 11 (Slc7a11) were decreased following CME, while that of Ptgs2 was significantly increased (Figures 1F–K). In addition, iron content and MDA levels were increased in the CME group compared to those in the sham group, while GSH accumulation was decreased (Figures 1M–1O). CME induced a decrease or even a disappearance of mitochondrial cristae and rupture of the outer mitochondrial membrane (Figure 1L). Notably, we also determined that levels of TNF-α and IL-1β, crucial markers of inflammation, were dramatically increased following CME (Figures 1P–T). These results indicate that ferroptosis and inflammation might be involved in CME-induced myocardial injury.

### Ferroptosis inhibition attenuated myocardial injury and inflammation following CME

**FIGURE 2:**
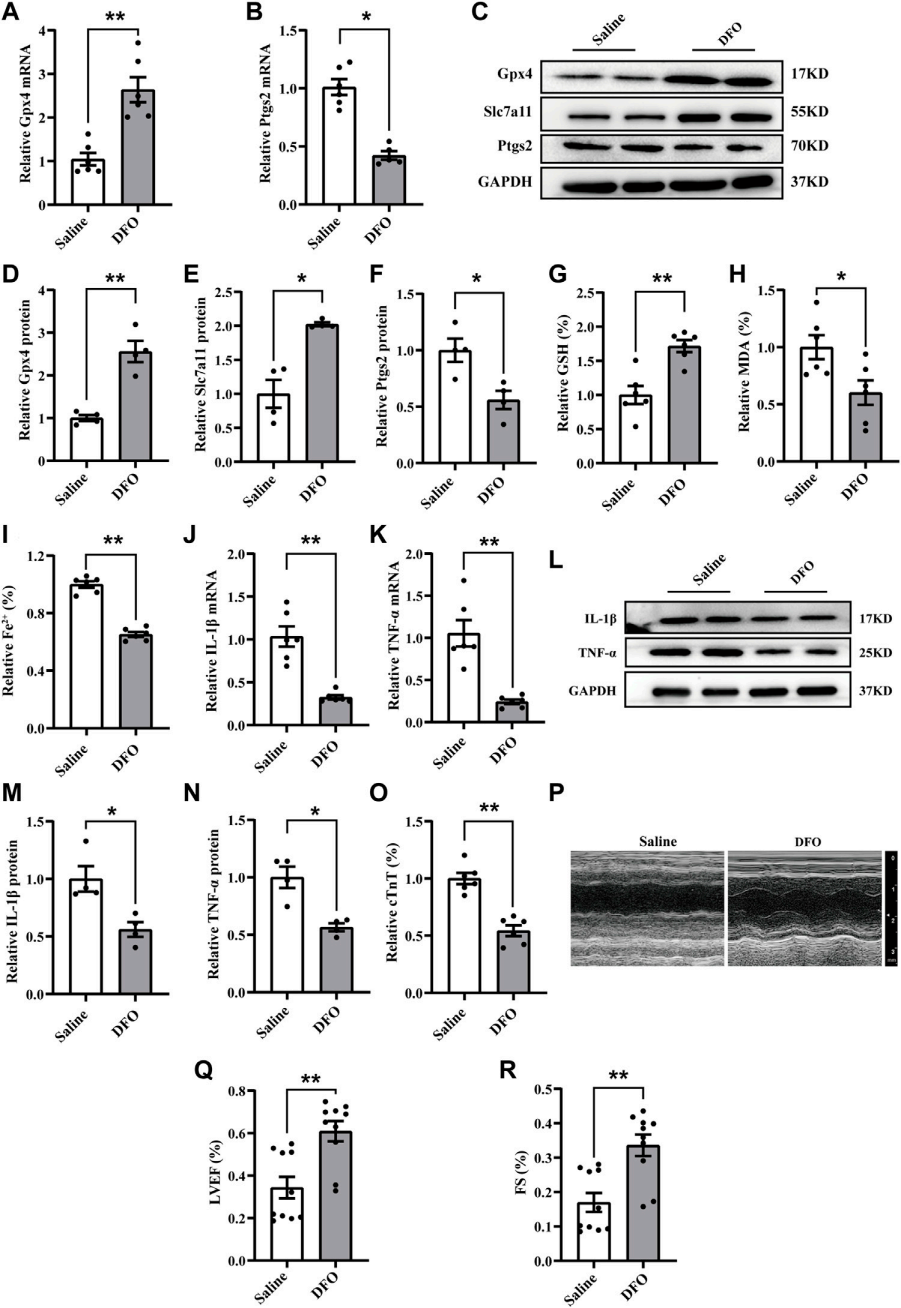
Ferroptosis inhibition attenuated myocardial injury and inflammation following CME. **(A,B)** The expression levels of Gpx4 and Ptgs2 mRNA (*n* = 6). **(C–F)** Western blotting showing the expression of Gpx4, Slc7a11, and Ptgs2 (*n* = 4). **(G–I)** Levels of GSH, Fe^2+^, and MDA in myocardial tissues (*n* = 6). **(J,K)** RT-qPCR was performed to quantify levels of IL-1β and TNF-α (*n* = 6). **(L–N)** Western blotting showing the expression of IL-1β and TNF-α (*n* = 4). **(O)** Serum levels of cTnT were decreased in the DFO group (*n* = 6). **(P–R)** Cardiac LVEF and FS detected by echocardiography (*n* = 10). GAPDH served as an internal control and was performed to quantitatively normalized the protein data. Data are presented as the normalized mean ± SEM (to Saline) or mean ± SEM. Values in salines were averaged and normalized to 1 **(A–O)**. *p < 0.05. **p < 0.01. CME: coronary microembolization; DFO: deferoxamine; LVEF: left ventricular ejection fraction; FS: fractional shortening; cTnT: cardiac troponin T; Gpx4: glutathione peroxidase 4; Slc7a11: solute carrier family 7 member 11; Ptgs2: prostaglandin-endoperoxide synthase-2; MDA: malondialdehyde; GSH: glutathione; IL-1β: interleukin 1 beta; TNF-α: tumor necrosis factor alpha.

To further verify the role of ferroptosis in CME-induced myocardial injury, deferoxamine (DFO), a well-known ferroptosis inhibitor, was administered. The results revealed that DFO pretreatment reversed the increased Ptgs2 and decreased levels of Slc7a11 and Gpx4 induced by CME (Figures 2A–F). Meanwhile, DFO reduced iron content and MDA levels and increased GSH accumulation (Figures 2G–I). The levels of TNF-α and IL-1β were also decreased after DFO administration (Figures 2J–N). Strikingly, DFO improved CME-induced myocardial dysfunction and decreased the level of cTnT (Figures 2O–R). These results confirmed the involvement of ferroptosis in CME-induced myocardial injury and inflammation.

### Ptgs2 silencing ameliorated ferroptosis-mediated myocardial injury and inflammation following CME

**FIGURE 3:**
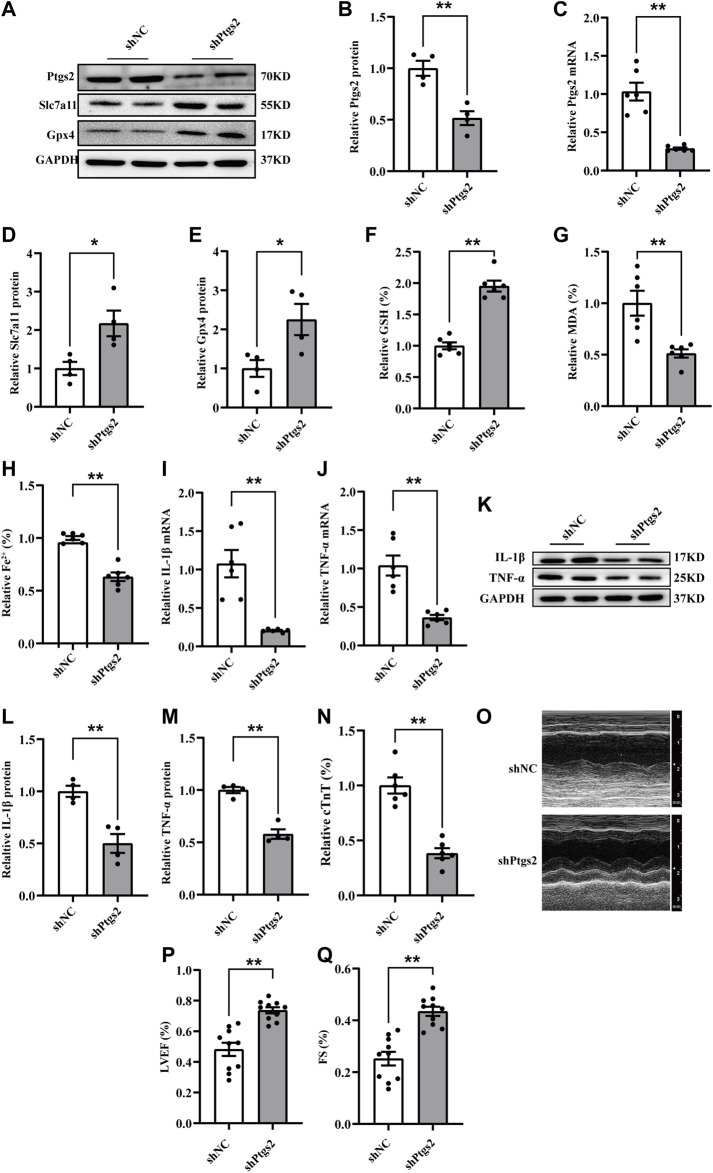
Ptgs2 silencing ameliorated ferroptosis-mediated myocardial injury and inflammation following CME. **(A–C)** The efficiency of Ptgs2 silencing in mRNA (*n* = 6) and protein (*n* = 4) level. **(D,E)** The expression levels of Gpx4 and Slc7a11 determined by western blotting (*n* = 4). **(F–H)** Levels of GSH, Fe^2+^, and MDA in myocardial tissues (*n* = 6). **(I,J)** The mRNA levels of TNF-α and IL-1β in each group (*n* = 6). **(K–M)** Western blotting showing the expression of IL-1β and TNF-α (*n* = 4). **(N)** Serum levels of cTnT were decreased in the shPtgs2 group (*n* = 6). **(O–Q)** Cardiac LVEF and FS detected by echocardiography (*n* = 10). GAPDH served as an internal control was performed to quantitatively normalized the protein data. Data are presented as the normalized mean ± SEM (to shNC) or mean ± SEM. Values in shNCs were averaged and normalized to 1 **(A–N)**.*p < 0.05. **p < 0.01. CME: coronary microembolization; LVEF: left ventricular ejection fraction; FS: fractional shortening; cTnT: cardiac troponin T; Gpx4: glutathione peroxidase 4; Slc7a11: solute carrier family 7 member 11; Ptgs2: prostaglandin-endoperoxide synthase-2; MDA: malondialdehyde; GSH: glutathione; IL-1β: interleukin 1 beta; TNF-α: tumor necrosis factor alpha.

To further investigate the regulatory mechanism of ferroptosis and inflammation following CME, we focused on the function of Ptgs2, a core factor of ferroptosis, in CME-induced myocardial injury. Considering the upregulation of Ptgs2 following CME, we transfected specific rAAV9 targeting Ptgs2 to inhibit its expression *in vivo*. The expression of Ptgs2 was effectively inhibited (Figures 3A–C). Ptgs2 knockdown reversed the decreased expression of Gpx4 and Slc7a11 following CME (Figures 3A,D,E). Meanwhile, the iron content and MDA level were decreased, and the GSH level was increased (Figures 3F–H). In addition, Ptgs2 inhibition significantly decreased the levels of TNF-α and IL-1β induced by CME (Figures 3I–M). Moreover, Ptgs2 silencing improved myocardial dysfunction and attenuated cardiac injury induced by CME (Figures 3N–Q). Taken together, these results indicated that Ptgs2 inhibition ameliorated ferroptosis-dependent myocardial injury and inflammation following CME.

### Hif1a positively regulated Ptgs2 by binding to its promoter region

**FIGURE 4:**
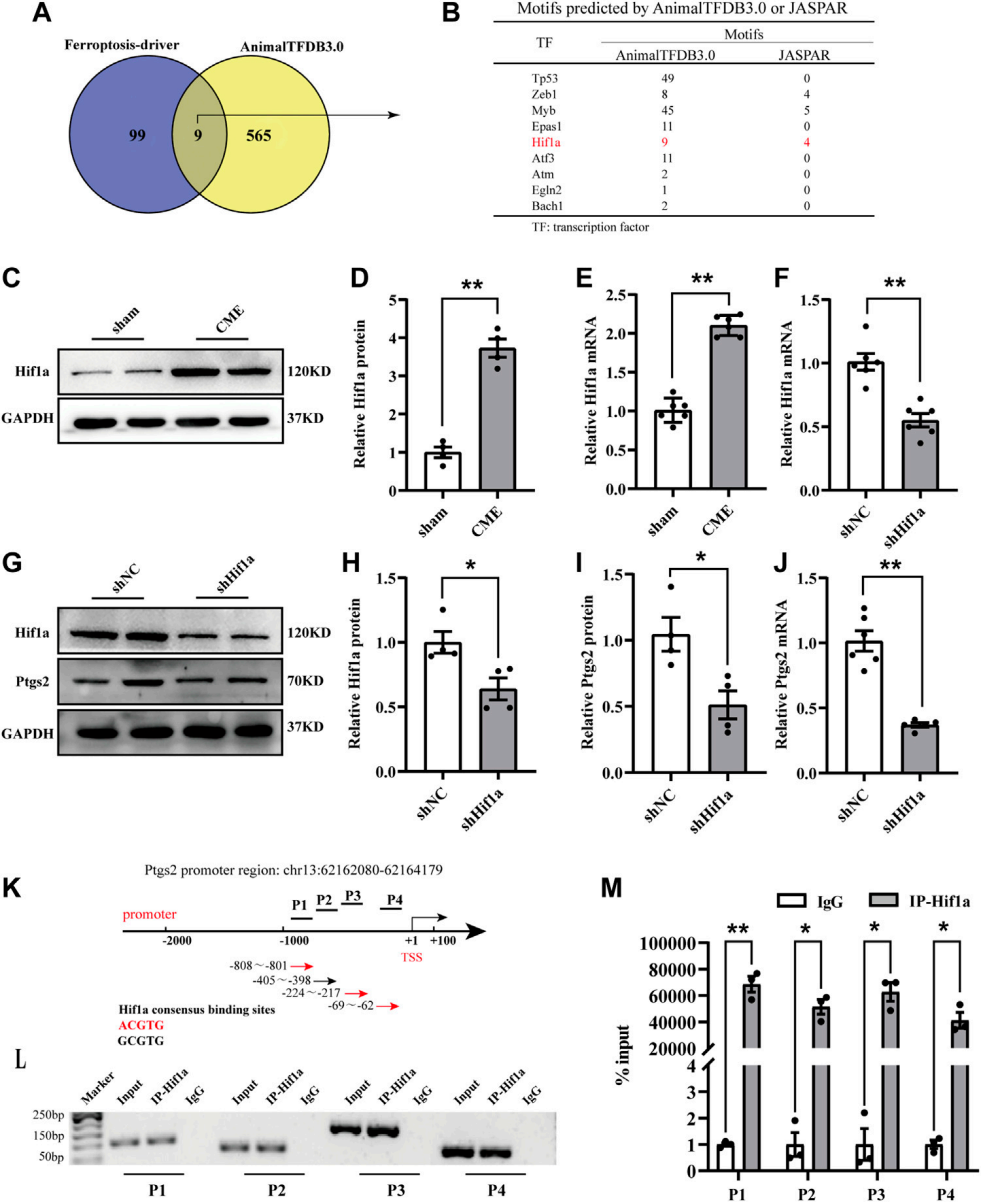
Hif1a positively regulated Ptgs2 by binding to its promoter region. **(A)** Veen digraph of candidate transcription factors determined by AnimalTFDB 3.0 intersecting with ferroptosis driver. Nine candidates were screened out. **(B)** Potential motifs of the nine candidates. **(C–E)** Hif1a expression following CME detected by RT-qPCR (*n* = 6) and western blotting (*n* = 4). **(F)** The expression level of Hif1a detected by RT-qPCR (*n* = 6). **(G,H)** The expression of Hif1a protein determined by western blotting (*n* = 4). **(I,J)** The expression of Ptgs2 determined by western blotting (*n* = 4) and RT-qPCR, respectively (*n* = 6). **(K–M)** ChIP-qPCR assay showing the interaction of Hif1a and four segments (P1-P4) of Ptgs2 promoter (*n* = 3). IgG was a negative control. GAPDH served as an internal control was performed to quantitatively normalized the protein data. Data are presented as the normalized mean ± SEM (to sham, shNC, or IgG). Values in shams, shNCs or IgGs were averaged and normalized to 1 **(C–M)**. *p < 0.05. **p < 0.01. Hif1a: hypoxia-inducible factor 1 subunit alpha; Ptgs2: prostaglandin-endoperoxide synthase-2; ChIP: Chromatin immunoprecipitation.

To clarify the transcriptional regulation of Ptgs2 following CME, we utilized the AnimalTFDB3.0 database to predict putative transcription factors (Hu et al., 2019). The results revealed that a total of 9 putative transcription factors were included by intersecting the results with the ferroptosis driver in FerrDb (Figures 4A,B) (Zhou and Bao, 2020). Among them, we were most interested in Hif1a. Hif1a was significantly upregulated following CME (Figures 4C–E). Strikingly, Hif1a silencing decreased the expression of Ptgs2 (Figures 4F–J). Then, JARSPA was used to predict putative motifs in the promoter region of Ptgs2 (Fornes et al., 2020). Notably, the results revealed that the Ptgs2 promoter region contains four Hif1a-binding elements, meaning that Ptgs2 is assumed to be a target of Hif1a (Figure 4B). The ChIP‒qPCR assay confirmed the binding of Hif1a to the promoter region of Ptgs2 (Figures 4K–M). These results indicated that Hif1a could bind to the promoter of Ptgs2 and positively regulate its transcription.

### ATV attenuated ferroptosis-dependent myocardial injury and inflammation following CME by inhibiting the Hif1a/Ptgs2 axis

**FIGURE 5:**
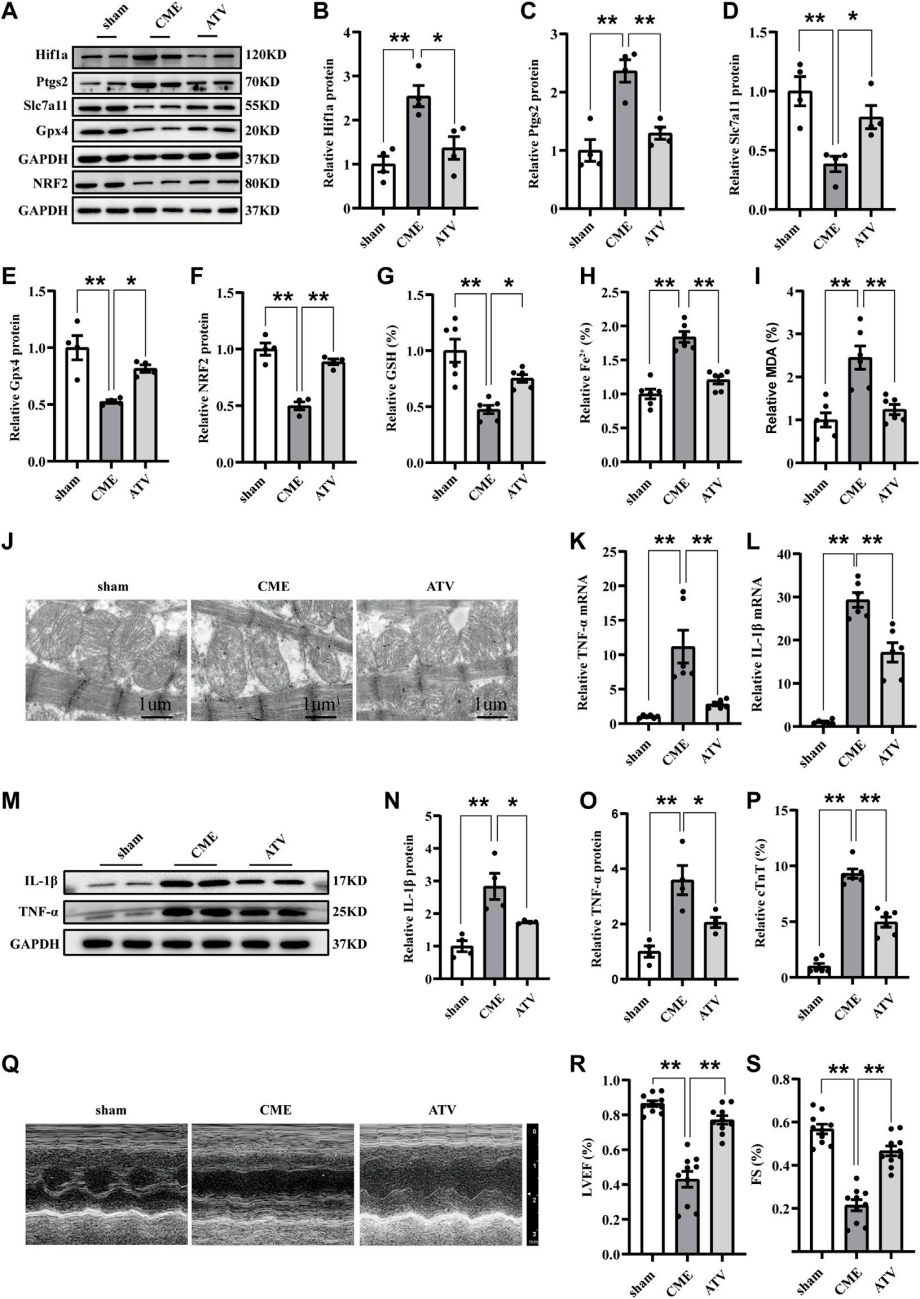
Atorvastatin attenuated ferroptosis-dependent myocardial injury and inflammation following CME by inhibiting Hif1a/Ptgs2 axis. **(A–F)** Western blotting was performed to determine the protein levels of Hif1a, Ptgs2, Gpx4, Slc7a11, and NRF2 (*n* = 4). **(G–I)** Levels of GSH, Fe^2+^, and MDA in myocardial tissues (*n* = 6). **(J)** Representative images of transmission electron microscopy. Scale bar = 1 μm. **(K,L)** RT-qPCR was used to detect the mRNA expression of IL-1β and TNF-α (*n* = 6). **(M–O)** Western blotting showing the expression of IL-1β and TNF-α (*n* = 4). **(P)** Serum levels of cTnT were detected in each group (*n* = 6). **(Q–S)** Cardiac LVEF and FS detected by echocardiography (*n* = 10). GAPDH served as an internal control was performed to quantitatively normalized the protein data. Data are presented as the normalized mean ± SEM (to sham) or mean ± SEM. Values in shams were averaged and normalized to 1 **(A–P)**. *p < 0.05. **p < 0.01. CME: coronary microembolization; Hif1a: hypoxia-inducible factor 1 subunit alpha; Ptgs2: prostaglandin-endoperoxide synthase-2; Gpx4: glutathione peroxidase 4; Slc7a11: solute carrier family 7 member 11; NRF2: nuclear factor erythroid 2-related factor 2; MDA: malondialdehyde; GSH: glutathione.

ATV has been demonstrated to exert anti-inflammatory and cardioprotective effects. We further investigated the function of Hif1a signaling in ATV-elicited protection against CME-induced myocardial injury. ATV pretreatment effectively inhibited the upregulation of Hif1a and Ptgs2 induced by CME (Figures 5A–C). Meanwhile, ATV increased the expression of Slc7a11, Gpx4, and nuclear factor erythroid 2-related factor 2 (Nrf2) following CME (Figures 5A,D–F). The iron content and MDA levels were lower in the ATV group than in the Saline group, and GSH levels were increased in the ATV group compared with the Saline group (Figures 5G–I). Moreover, ATV pretreatment significantly ameliorated mitochondrial damage (Figure 5J). In addition, ATV reduced the levels of TNF-α and IL-1β induced by CME (Figures 5K–O). ATV improved myocardial dysfunction and reduced cardiac injury following CME (Figures 5P–S). Taken together, these results indicate that ATV attenuates ferroptosis-dependent myocardial injury and inflammation following CME by inhibiting the Hif1a/Ptgs2 axis.

## Discussion

In the present study, we found that ferroptosis and inflammation were involved in CME-induced myocardial injury and that DFO pretreatment reversed this effect. Ptgs2, positively regulated by Hif1a, contributed to cardiac ferroptosis and the inflammatory response following CME. In addition, ATV ameliorated ferroptosis-dependent myocardial injury and inflammation following CME by inhibiting the Hif1a/Ptgs2 pathway.

Ferroptosis is a newly identified form of regulated cell death that is characterized by iron-dependent accumulation of lipid peroxidation and inactivation of the antioxidant system (Stockwell, 2022). In the past 10 years, ferroptosis has increasingly become a hotspot for investigating the progression of cardiovascular diseases (Fang et al., 2022). Fang et al. (2019) showed that ferrostatin-1, a specific ferroptosis inhibitor, attenuated myocardial dysfunction induced by both acute and chronic myocardial ischemia-reperfusion injury. Coronary microvascular dysfunction is a significant feature of patients with diabetes. These patients have a worse prognosis after cardiac ischemia events than those without diabetes (Horton and Barrett, 2021). In 2022, ferroptosis was demonstrated for the first time in the heart of a mouse model with diabetic cardiomyopathy, in which NRF2 activation protects against ferroptosis-dependent diastolic dysfunction (Wang et al., 2022). Combined with our results, these data validate the vital role of ferroptosis in the progression of cardiovascular diseases. Notably, our team and others previously demonstrated that apoptosis and pyroptosis were involved in myocardial dysfunction following CME (Liu et al., 2015; Zhou et al., 2021). Considering the differences in their morphological changes and downstream signaling pathways, further investigations into the underlying mechanisms of the interaction modes among these forms of regulated cell death are needed.

Increasing evidence demonstrates that ferroptosis plays a proinflammatory role, and several ferroptosis inhibitors have been determined to exert anti-inflammatory effects (Sun et al., 2020). Li et al. (2019) revealed that ferrostatin-1 inhibited cardiomyocyte cell death and blocked the recruitment of neutrophils after heart transplantation. Furthermore, researchers have demonstrated that ferroptosis promotes the recruitment of neutrophils to the injured myocardium and thus induces an inflammatory response. In addition, Yu et al., found that acyl-CoA synthetase long-chain family member 4 (ACSL4), a pivotal and valid ferroptosis driver, aggravated ischemic stroke by promoting ferroptosis-dependent brain injury and neuroinflammation. Importantly, ACSL4 silencing inhibited the accumulation of proinflammatory cytokines, including TNF-α, IL-1β, and IL-6 (Cui et al., 2021). Consistent with these results, we found that the inflammatory response was accompanied by ferroptosis events following CME, and ferroptosis inhibition by DFO inhibited the increase in proinflammatory cytokines induced by CME. Further research is needed to provide evidence on the mechanism of the interaction between ferroptosis and inflammation. Taken together, these results suggest that ferroptosis plays a proinflammatory role.

Ptgs2 is usually recognized as a marker of ferroptosis (Tang et al., 2021). However, Xiao et al., revealed that miR-212-5p attenuated neuronal ferroptosis following traumatic injury by targeting Ptgs2 (Xiao et al., 2019). Whether Ptgs2 contributes to ferroptosis remains controversial. In the present study, we found that Ptgs2 was significantly increased following CME. Ptgs2 inhibition reversed the increases in ferroptosis and inflammation following CME, indicating that Ptgs2 may mediate cardiac ferroptosis and inflammation following CME. Hif1a can either promote or inhibit the transcription of prosurvival genes under different pathological conditions and thus plays a bidirectional role in the regulation of cellular functions and fates (Choudhry and Harris, 2018). Wu et al. (2019) revealed that Hif1a promoted hypoxia-induced cardiomyocyte apoptosis by inhibiting the expression of miR-10b-5p. Meanwhile, Hif1a has been recognized as a proinflammatory factor (Palazon et al., 2014). Our results showed that Hif1a expression was significantly increased following CME, and highly expressed Hif1a positively regulated Ptgs2 expression by binding to its promoter region.

The benefit of long-term statin treatment in decreasing major cardiovascular events mainly relies on the reduction of low-density lipoprotein. The clinical significance of the pleiotropic effects of statins in the cardiovascular system remains controversial (Oesterle et al., 2017). Mechanistic studies have suggested that statins exert anti-inflammatory and antioxidant effects. Given the involvement of lipid peroxidation in ferroptosis, it is easy to understand the effect of ATV on ferroptosis inhibition. Ning et al., found that ATV attenuated myocardial dysfunction and remodeling following isoproterenol challenge through the inhibition of ferroptosis (Ning et al., 2021). In the present study, we demonstrated that ATV significantly improved myocardial function following CME by inhibiting ferroptosis and inflammation *via* the Hif1a/Ptgs2 pathway.

## Conclusion

In conclusion, we determined that Ptgs2 was positively regulated by Hif1a and contributed to ferroptosis-dependent myocardial injury and inflammation. Furthermore, ATV protects against ferroptosis and inflammation induced by CME *via* the Hif1a/Ptgs2 pathway.

## Data Availability

The original contributions presented in the study are included in the article/Supplementary Material, further inquiries can be directed to the corresponding author.
